# Editorial: Natural products and pseudo-natural products against veterinary disease-causing microorganisms

**DOI:** 10.3389/fvets.2024.1429587

**Published:** 2024-05-27

**Authors:** Muhammad Mohsin, Muhammad Tahir Aleem, Mohsan Ullah Goraya, Liliana Aguilar-Marcelino, Rao Zahid Abbas, Asghar Abbas

**Affiliations:** ^1^Department of Pharmacology, Shantou University Medical College, Shantou, Guangdong, China; ^2^School of Medicine, Huaqiao University Quanzhou, Quanzhou, China; ^3^Centro Nacional de Investigación Disciplinaria en Salud Animal e Inocuidad, Instituto Nacional de Investigaciones Forestales, Agrícolas y Pecuarias (INIFAP), Mexico, Mexico; ^4^Department of Parasitology, University of Agriculture, Faisalabad, Pakistan; ^5^Department of Pathobiology and Biomedical Sciences, Muhammad Nawaz Shareef University of Agriculture Multan, Multan, Pakistan

**Keywords:** natural products, novel compounds, diseases control measures, veterinary drug, drug development

The livestock/poultry sector plays a vital role in the economy, particularly in developing countries, contributing significantly to the Gross Domestic Product (GDP) and addressing key agricultural challenges. However, its growth is hindered by the prevalence of viral, bacterial, parasitic, and fungal infections, which cause significant morbidity and mortality among livestock/poultry populations. These diseases have devastating effects on animal health and impose substantial financial burdens on the livestock/poultry industry (1). Moreover, the sector faces challenges related to developing diseases attributed to synthetic antibiotics and drugs in animal feed. As a response, European nations have taken stringent measures by completely banning synthetic antibiotics.

Consequently, exploring innovative treatment options for preventing infectious diseases has become an intriguing strategy (1). Studies have demonstrated the potential effectiveness of nanoparticles and plant-based products in combating pathogens (2). Essential oils and botanical substances have also shown notable effectiveness against infectious diseases. Phytochemicals have demonstrated favorable results against pathogens such as *Salmonella typhimurium* (3, 4) (Almuzaini; Liu et al.; Mao et al.). Chlorogenic acid has emerged as a promising drug for the treatment and control of *Riemerella anatipestifer*. Icariin, derived from herbs, has exhibited positive effects against cisplatin-induced renal cell damage in canines (Liu et al.). Probiotics have proven to be beneficial ingredients in animal feed. In treatment alone and combination with novel plants and prebiotics, probiotics have emerged as a viable alternative to antibiotics. The role of lactic acid bacteria as antioxidants and anti-inflammatories makes them effective against *Staphylococcus aureus* as proven in different studies (Mao et al.) (3).

Overall, this Research Topic offers a brief overview of the current research landscape concerning novel compounds and advancements in controlling infectious diseases in livestock and poultry. We anticipate that the findings presented in this topic will enhance our understanding and introduce new strategies for effectively managing various infectious diseases.

## Author contributions

MM: Conceptualization, Writing—original draft. MA: Resources, Writing—review & editing. MG: Writing—review & editing, Data curation. LA-M: Writing—review & editing, Formal analysis. RA: Writing—review & editing, Supervision. AA: Writing—review & editing, Formal analysis.
